# Antimicrobial Nanoparticles Composed of Zein and Arginine-Phenylalanine-Based Surfactants for Wound Related Infections: Antioxidant and Skin-Related Anti-Enzymatic Activities and Toxicity

**DOI:** 10.3390/antibiotics13121149

**Published:** 2024-12-01

**Authors:** Francisco Fábio Oliveira de Sousa, Zakaria Hafidi, María Teresa García, Maria del Carmen Moran, Sergio Vazquez, Lourdes Pérez

**Affiliations:** 1 Laboratory of Quality Control, Bromatology & Microbiology, School of Pharmacy, Department of Biological & Health Sciences, Federal University of Amapá, Rodovia Juscelino Kubitscheck, km 02, Macapa 68903-419, Brazil; 2 Department of Surfactants and Nanobiotechnology, Instituto de Química Avanzada de Cataluña, Centro Superior de Investigaciones Científicas IQAC-CSIC, 08035 Barcelona, Spain; zhatnt@cid.csic.es (Z.H.); teresa.garcia@iqac.csic.es (M.T.G.); svbtlb@cid.csic.es (S.V.); 3 Secció de Fisiologia, Departament de Bioquímica i Fisiologia, Facultat de Farmàcia i Ciències de l’Alimentació, Universitat de Barcelona, Avda. Joan XXIII 27-31, 08028 Barcelona, Spain; mcmoranb@ub.edu; 4 Institut de Nanociència i Nanotecnologia—IN2UB, Universitat de Barcelona, Avda. Diagonal, 645, 08028 Barcelona, Spain

**Keywords:** arginine, phenylalanine, surfactants, zein, nanoparticles, enzymes, skin

## Abstract

**Background/Objectives**: Cationic surfactants are potential antimicrobial candidates. Even so, they are the foremost irritative and incompatible group, which limits their usage. The incorporation of surfactants in biopolymer-based nanoparticles is a feasible strategy to improve their efficacy and reduce those drawbacks. **Methods**: Surfactants with one amino acid on the polar head (lauroyl arginine methyl ester—LAM and phenylalanine dodecyl amide—PNHC_12_) and surfactants with two amino acids on the polar heads, arginine-phenylalanine (Lauroyl phenylalanine arginine methyl esther—C_12_PAM and phenylalanine-arginine dodecyl amide—PANHC_12_) were loaded to zein nanoparticles. Their antimicrobial and antibiofilm activities were evaluated. Also, the inhibitory activities of the surfactants and nanoparticles over skin-related enzymes were accessed in silico and in vitro, while their cytotoxicity was determined comparatively over immortal human keratinocytes (HaCaT) and human fibroblasts (3T3). Finally, the *Vibrio fisheri* luminescence reduction test was used to detect its ecotoxicity. **Results**: The nanoparticles were obtained successfully and exhibited good biocide activity against a wide range of pathogenic bacteria and yeasts. The surfactants were found active over the enzymes assayed: elastase > tyrosinase > collagenase > lipoxygenase, while the inhibitory activity was superior when nanoencapsulated over the enzymes tyrosinase and lipoxygenase. The surfactants and their corresponding nanoparticles presented acceptable cytotoxic levels, except for PNHC_12_ in both forms, while their ecotoxicity was limited and acceptable. **Conclusions**: Accordingly, the nanoencapsulation of the arginine-phenylalanine surfactants loaded to zein nanoparticles was found to be a smart strategy to enhance the antimicrobial activity and improve their selectivity over representative skin and connective tissues cell lines. These biological properties render the arginine-phenylalanine surfactant nanoparticles as promising candidates for antimicrobial and tissue repairing applications in wound treatments.

## 1. Introduction

Surfactants are versatile agents used in pharmaceutical products. They can be both active ingredients and formulation adjuvants. Cationic surfactants are widely known for their superior antimicrobial properties [1]. Aminoacidic-based surfactants have gained attention over the last years, as they are more biocompatible and biodegradable than other groups [2]. However, they still keep some irritative and incompatible aspects for skin and wound care applications.

**Figure 1:**
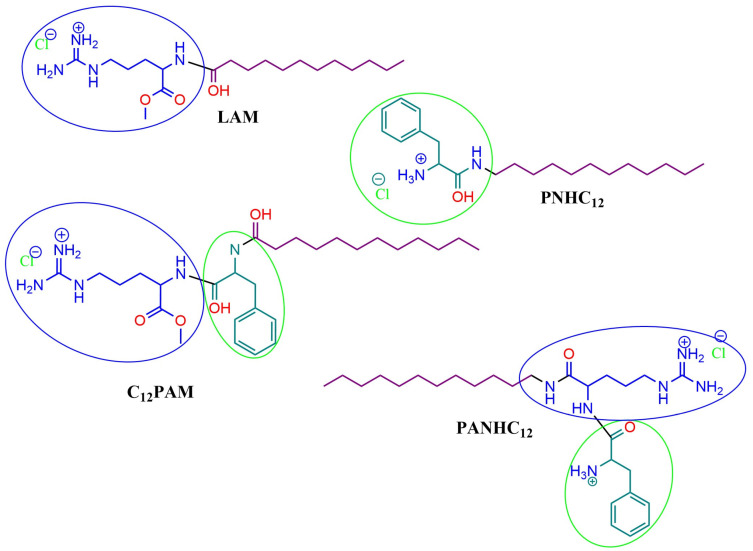
Chemical structures of N^α^-Lauroyl Arginine methyl ester (LAM), Phenylalanine lauroyl amide (PNHC_12_), N^α^-Lauroyl Phenylalanine Arginine methyl ester (C_12_PAM), and Phenylalanine Arginine lauroyl amide (PANHC_12_).

Novel surfactants containing in the polar head both arginine and phenylalanine have recently been synthesized [3]. The structures and acronyms of the new surfactants are shown in Figure 1. Note the difference in grouping positions of the combined arginine-phenylalanine surfactants. Therefore, this aspect could modulate their biological activities. In our previous study [3], these surfactants were found to exhibit good antibacterial and antifungal inhibitory activities.

Recently, we have formulated surfactant-based nanoparticles with these amino acid-based surfactants and found that this strategy improved their stability and enhanced their antimicrobial activity [3,4]. Nonetheless, some knowledge over their biocide and antibiofilm activities, enzymatic modulation, and cytotoxicity were not explored. In the course of skin damage, such as those produced by infections, the surrounding tissues are easily affected and destroyed. Although the elimination of the bacteria is challenging and a foremost goal in anti-infectious treatments [5], protecting and recovering the morphophysiological characteristics of the affected tissues is also a primary need. Therefore, associating both antimicrobial and repairing properties in the active ingredients is a great challenge and a foremost goal in anti-infectious treatments.

Some proteolytic enzymes are involved in the mechanisms of skin and connective tissue repair. For example, collagenase and elastase are responsible for degrading collagen and elastin fibers in the extracellular matrix [5].

Tyrosinase is enrolled in the melanin production and its regulation is important to guarantee skin uniformization. Lipoxygenase is involved in the inflammatory responses [6]. Considering these characteristics, the effect of these amino acid-based surfactants in the inhibition of these four enzymes was determined to explore the possible applications of these compounds for skin repairing, such as wound treatments.

## 2. Results and Discussion

### 2.1. Nanoparticles Characteristics

The nanoparticles containing the surfactants were obtained successfully. They were found to be spherical, uniform, monodispersed, and stable at room temperature. The main characteristics are displayed in Table 1. All formulations presented pdI values < 0.3, indicating a monodisperse system. The zeta potential of the blank zein nanoparticles (BZNps) was found positive, agreeing with our previous studies [3,4]. Moreover, the nanoparticles containing the surfactants presented higher zeta potential values than BZNps, while those containing the surfactant with two positive charges (NpPANHC_12_) presented even higher zeta potential values.

**Figure 2:**
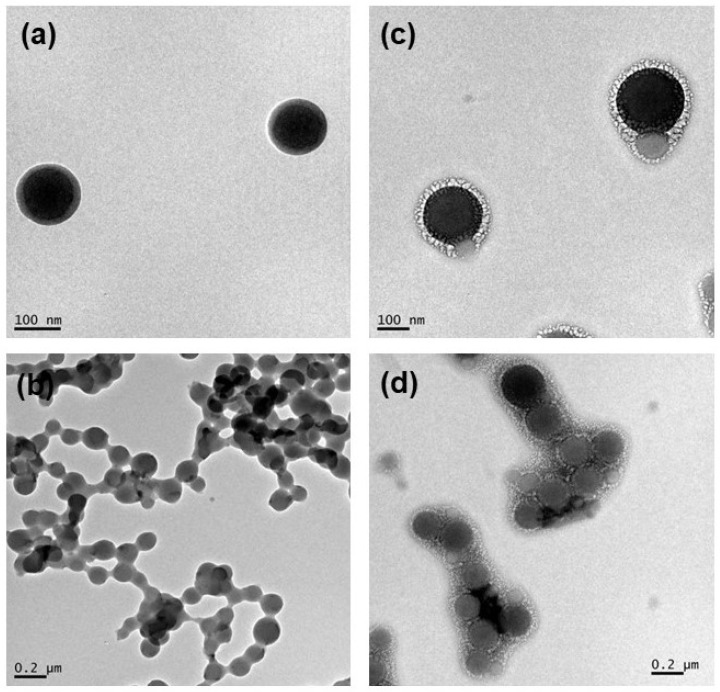
Transmission electronic microscopy images of (**a**,**b**) blank and (**c**,**d**) Lauroyl arginine methyl ester (LAM) loaded-zein nanoparticles under different magnifications.

The microscopic appreciation of the colloidal systems showed a different aggregation behavior after the incorporation of the surfactants to zein nanoparticles. While the BZNp was found to be monodisperse and presented a spherical, smooth, and uniform surface, the nanoparticles containing the surfactants presented a covering layer uniformly dispersed in their surface (Figure 2). This aspect can affect directly in the system stability and also on the resulting biological aspects discussed ahead.

**Figure 3:**
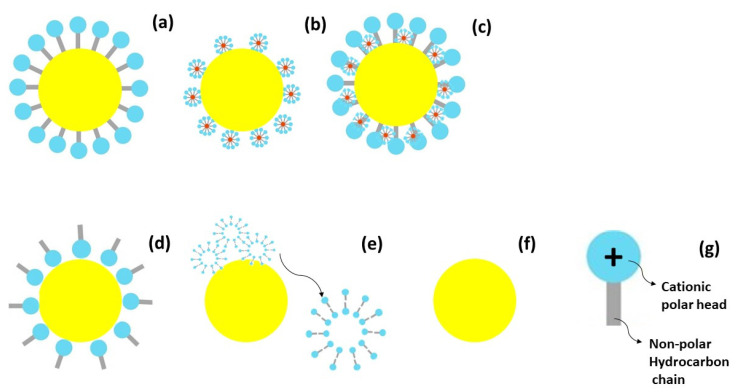
Aggregation possibilities for zein nanoparticles loaded with arginine-phenylalanine surfactants. (**a**) The hydrophobic hydrocarbon chains are directly connected to the polymeric nanostructure, (**b**) nanomicelles with hydrophobic core formed by the surfactants are in contact with the polymeric structure, (**c**) combination of direct interaction of the surfactants with nanomicelles interactions, (**d**) surfactants’ polar heads are connected to the polymeric structure or (**e**) larger micelles or bilayers containing water are formed and agglomerate to the polymeric nanoparticles structure. (**f**) Blank zein nanoparticles and (**g**) the general cationic surfactant chemical structure.

The physicochemical characteristics aspects (Table 1), together with the microscopic appreciation indicate that the surfactants may have followed different chemical conformations while loading to zein and to the nanometric structures obtained, such as illustrated in Figure 3. Zein, due to its amphiphilic characteristics, presents self-assembly properties. Self-assembly is a process where a disordered system forms an organized structure without support or orientation from external agents. It depends on weak chemical bonds, such as van der Waals, capilar, π-stacking, and hydrogen bonds [7]. Wang et al. [8] studied the microphase formation of zein in ethanol/water. Both spheres and lamellar phases were observed after the increment of zein concentration in the samples analyzed. Surfactants, as amphiphilic molecules, tend to form micelles or coating surfaces, according to their structure or associated charge. In view of the mono- or di-cationic molecules used in the nanoparticles’ formations, and the possibilities to bind through one or two of these charges, the surfactants can present different conformation possibilities, such as those proposed and demonstrated in previous studies [5]. These molecules can attach directly over zein nanoparticles, or blebs may grow and attach around the nanostructures. In both cases, they may interact through the hydrophilic or hydrophobic portions, considering, for instance, the predilection affinity of zein through aromatic rings [9], which are more likely to bind directly to the aromatic amino acids, such as phenyalanine, as previously described [3]. For the non-aromatic surfactants (such as LAM), the blebbing effect is more likely to occur with high accumulation of molecular aggregates. Higher zein concentrations resulted in a lamellar phase and film formation. In contrast, lower concentrations resulted in spherical formations, similar to those obtained in the present work.

### 2.2. Antimicrobial Activity

**Figure 4:**
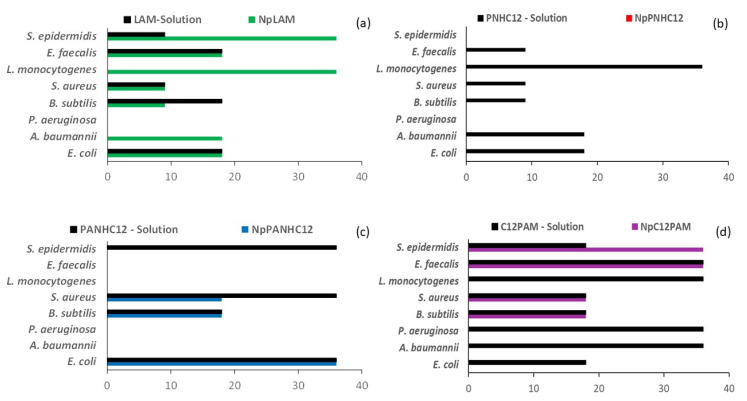
Minimum bactericide concentrations (MBC) (in µg/mL) of the surfactants in solution and nanoparticles: Lauroyl Arginine methyl ester (LAM) (**a**), Phenylalanine lauroyl amide (PNHC_12_) (**b**), Phenylalanine Arginine lauroyl amide (PANHC_12_) (**c**), and Lauroyl Phenylalanine Arginine methyl ester (C_12_PAM) (**d**). Concentrations assayed: 2.2, 4.4, 8.9, 17.8, and 35.6 µg/mL.

**Figure 5:**
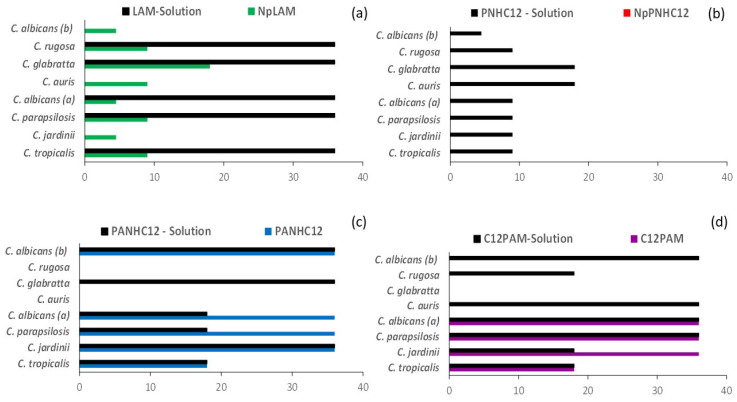
Minimum fungicide concentrations (MFC) (in µg/mL) of the surfactants in solution and nanoparticles: Lauroyl Arginine methyl ester (LAM) (**a**), Phenylalanine lauroyl amide (PNHC_12_) (**b**), Phenylalanine Arginine lauroyl amide (PANHC_12_) (**c**) and Lauroyl Phenylalanine Arginine methyl ester (C_12_PAM) (**d**). Concentrations assayed: 2.2, 4.4, 8.9 17.8 and 35.6 µg/mL.

The minimum bactericidal/fungicide (MBC/MFC) concentrations for the surfactants and their respective nanoparticles against representative Gram-positive, Gram-negative bacteria and yeasts are displayed in Figure 4 and Figure 5, respectively. The surfactants showed good bactericidal and fungicidal activity against a wide range of microorganisms. The MBC/MFC values are similar to the MIC values recently reported [3]. The activity depended greatly on their molecular structure, specifically their polar head. Our results indicate that these amino acid-based surfactants exhibited a superior activity against the Gram-positive bacteria, a common behavior for antimicrobials exhibiting a detergent-like mechanism [2], where they attack primarily the microbial membranes. The composition of the bacterial membrane of the Gram-negative bacteria hampers the interaction of surfactants with the cellular membranes [10]. Previous studies showed that the mechanism of action of cationic surfactants involves the electrostatic interaction between the charges of the protonated cationic groups and the negative charges located in the microbial membranes, followed by the hydrophobic interaction of the alkyl chains in the intramembrane region [1]. Therefore, the development of a resistance mechanism is more unlikely. The high pKa values of the guanidine group ensure that these compounds carry at least one cationic charge at physiological pH, a key feature for damaging bacterial membranes [2]. The LAM solution was the most effective formulation against all tested bacteria and fungi, demonstrating strong activity against both Gram-positive and Gram-negative planktonic bacteria, including some problematic pathogens such as *L. monocytogenes* and *P. aeruginosa* (Figure 4) and *C. auris* (Figure 5) that are commonly resistant to several antimicrobial agents. Its good solubility and the presence of arginine group may have played an important role in the result obtained. In contrast, while the PNHC_12_ surfactant was found to be very active in solution, its nanoencapsulation in zein nanoparticles abolished its activity over bacteria and yeasts (Figure 4 and Figure 5, respectively). One of the possible reasons for this phenomenon is the affinity of the aromatic phenylalanine groups to zein, such as reported in our previous study [3]. Therefore, considering this affinity together with its small molecular size, it may have been unavailable to act properly over the microorganisms. Moreover, the surfactants containing the two amino acids, i.e., arginine and phenylalanine (PANHC_12_ and C_12_PAM), presented a moderate antimicrobial activity, which was maintained after their nanoencapsulation in zein nanoparticles (Figure 4 and Figure 5).

### 2.3. Antibiofilm Activity

Nowadays, bacterial and fungal biofilms are implicated in most hospital-acquired infections and are one of the key virulence factors promoting the growth of resistant bacteria and fungi [11]. Once the biofilm is formed and matured, it becomes very difficult to eliminate due to the presence of a polysaccharide-based matrix [5]. Sessile cells are 10- to 1000-fold more resistant than their planktonic counterparts. Therefore, it is essential to develop effective antimicrobial and antibiofilm strategies

**Figure 6:**
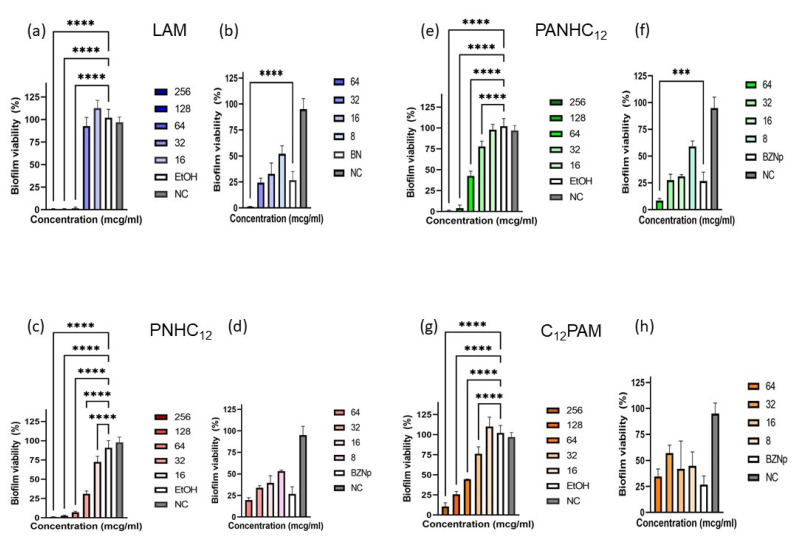
Antibiofilm activity of the surfactant-loaded zein nanoparticles: LAM solution (**a**) and nanoparticles (**b**), PNHC_12_ solution (**c**) and nanoparticles (**d**), PANHC_12_ solution (**e**) and nanoparticles (**f**), and C_12_PAM solution (**g**) and nanoparticles (**h**) over Methicillin-resistant *Staphylococcus aureus* (MRSA). (*** and **** mean *p* < 0.005 and *p* < 0.001, respectively).

**Figure 7:**
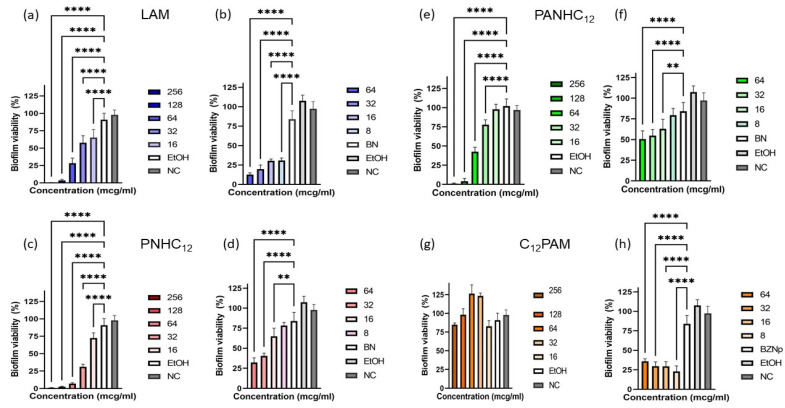
Antibiofilm activity of the surfactants-loaded zein nanoparticles: LAM solution (**a**) and nanoparticles (**b**), PNHC12 solution (**c**) and nanoparticles (**d**), PANHC_12_ solution (**e**) and nanoparticles (**f**), and C_12_PAM solution (**g**) and nanoparticles (**h**) over *Candida albicans*.(** and **** mean *p* < 0.01 and *p* < 0.001, respectively).

In view of the favorable biocide results over the microorganisms in suspension, the surfactants solutions and nanoparticles were tested over MRSA and *C. albicans* biofilms, two of the most common strains found in chronic wounds. Preformed biofilms of these two microorganisms were treated with different concentrations of both the surfactants (256–16 µg/mL) in the ethanol/water solutions and nanoparticles (64–8 µg/mL) with and without surfactants. After 24 h, the percentage of biofilm eradication was measured by MTT staining assay (Figure 6 and Figure 7). The susceptibility of MRSA to pure surfactant solutions depends on the amino acids present on their polar head. Surfactants with a single amino acid exhibited the best ability to disperse the MRSA biofilms. For instance, LAM and PNHC_12_ were able to fully eradicate the biofilms at 64 µg/mL while, at this concentration, C_12_PAM and PANHC_12_ only removed around 60%. Figure 6 also shows the antibiofilm activity of the blank NPs; it can be observed that these formulations also dispersed a high percentage of biofilm, around 70% at 64 µg/mL. The antibiofilm efficiency of the NPs containing these surfactants seem to be the sum of the pure surfactants with that of the blank NPs (Figure 6 and Figure 7) except for the C_12_PAM NPs; this formulation did not show antibiofilm activity. These results are consistent with the tendencies observed in the MBC/MFC values, except for NpPNHC_12_, which presented a good antibiofilm activity, unlike the biocide tests.

Notice that in this work we have evaluated biofilm eradication and not biofilm inhibition. Usually, higher concentrations are needed to eradicate mature biofilms because the required mechanism is more complicated, given that surfactants have to disrupt the extracellular polymeric matrix and kill the bacteria. For NP formulations, their physicochemical properties (particle size, zeta potential) are critical for biofilm eradication. The size affects the diffusion of the antimicrobial through the exopolysaccharide matrix. It seems that NPs with a size between 10 and 500 nm can better penetrate through the water channels and biofilm pores [12,13]. The average diameter of the NPs used in this work ranged from 194 to 312 nm, as such, it is expected that they can easily penetrate into the biofilms. Moreover, these NPs exhibited a high positive zeta potential, between +25 and +44 mV. This is another important parameter that can improve the biofilm penetration, given that positively charged aggregates are more attracted to negatively charged biofilm surfaces [14]. Furthermore, it is very interesting that the biofilm disruptors also have antimicrobial properties, as they can both detach the bacteria from the polymeric matrix and eliminate them. Given the good properties of zein, several works regarding the preparation of NPs with antimicrobial compounds can be found in the literature: (1) ZNPs containing ceftazidime and tobramycin showed very good inhibition and eradication of biofilms [15], (2) the antibiofilm and anti-virulence activities of phytochemicals, such as curcumin [16] and anacardic and ellagic acids were improved by its encapsulation in zein nanoparticles, and (3) the co-encapsulation of ceftazidime and tobramycin in zein nanoparticles coated with chitosan showed enhanced antibacterial and antibiofilm activities compared to the individual formulations [17]. However, to our knowledge, only our group has recently studied zein nanoparticles containing antimicrobial biocompatible amino acid-based surfactants [3,4].

The results concerning the antibiofilm activity against *C. albicans* are shown in Figure 7. The ratio between the activity of free solution/encapsulated NPs follows a similar pattern to that observed against MRSA. The most active surfactant was PNHC_12_, which was found to almost remove totally the biofilm at 64 µg/mL. In contrast, the least active surfactant was C_12_PAM, which at the same concentration did not cause any disruption to the biofilm. LAM and PANHC_12_ exhibited a similar effectiveness. The activity of C_12_PAM was significantly improved after its nanoencapsulation in zein nanoparticles; this surfactant did not removed biofilm at any of the tested concentrations, while its NPs were able to disperse around the 70% of biofilm. The efficiency of LAM also improved slightly, while for the other two surfactants, the activity of the NPs was found to be similar to that shown by the solutions.

### 2.4. Antioxidant Activity

**Figure 8:**
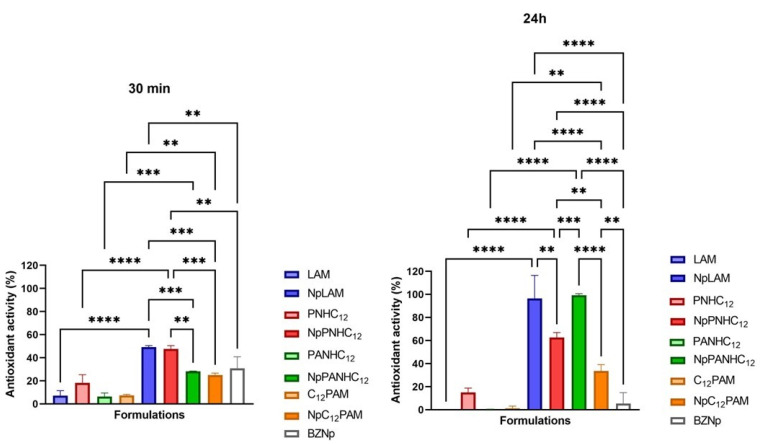
Antioxidant activity of amino acid-based surfactants (LAM, PNHC_12_, C_12_PAM, and PANHC_12_) and their corresponding Nps at 35.6 µg/mL after 30 min and 24 h. (**, *** and **** mean *p* < 0.01, *p* < 0.005 and *p* < 0.001, respectively).

The imbalance of the equilibrium between ROS (reactive oxygen species) generation and the antioxidant systems can raise the aging processes and cause several diseases. In this work the free radicals’ scavenging ability of the surfactant solutions and the loaded NPs have been evaluated using the DPPH scavenging radical test (Figure 8). This test is widely used to evaluate the antioxidant activity of organic compounds. The formulations were mixed with DPPH and checked after 30 min and 24 h. The surfactants solely presented a limited antioxidant activity, which was abolished after 24 h (Figure 8), while at 30 min blank NPs scavenged the 30% of free radicals. The presence of leucine, proline, and histidine in the zein structure permits proton donation to radicals that are deficient in electrons and, thus, it can enhance the inhibition of free radicals [18]. After 30 min, the antioxidant effect of the loaded NPs seems to be the sum of the antioxidant effect of NPs and the amino acid-based surfactants loaded to zein. However, after 24 h, the antioxidant activity of zein nanoparticles containing the surfactants was clearly superior to the sum of the of both components individually. Therefore, nanoencapsulation of the surfactants is a promising strategy to combine the stabilization of zein nanoparticles and reach a synergic antioxidant effect. In some cases, a sustained antioxidant activity can be observed, especially when controlled release systems are assayed. For instance, zein nanoparticles have demonstrated this aspect before when polyphenols were nanoencapsulated. It may come from the fast antioxidant activity of the solutions in contrast to the nanoparticles, which can possibly inhibit the DPPH radical in a more sustainable manner. The long-term antioxidant activity of these NPs can help to maintain the balance between ROS production and the antioxidant system and if so, reduce the skin damage by aging. This aspect is also important to formulate novel products containing these formulations as they could extend the time of application considering the sustained activity found.

Due to its biocompatibility and their intrinsic antioxidant activity, zein has been widely used to encapsulate antioxidant active molecules. In some cases, the nanoencapsulation of these active molecules reduces their radical inhibition capacity. For example, it has been reported that free resveratrol presented a higher ability in the inhibition of ABTS radicals than nanoencapsulated RVT [19] and zein nanoparticles containing resveratrol, and tocopherol also showed this behavior [20]. However, other studies showed that the nanoencapsulation procedure enhanced the antioxidant efficiency of the bioactive molecules [21].

### 2.5. Anti-Enzymatic Inhibitory Activities

The inhibitory activities of the surfactants and the loaded NPs over collagenase, elastase, tyrosinase, and lipoxygenase, key enzymes enrolled in the tissue repairing processes, were evaluated. Collagen, elastin, and tyrosine are some of the proteins affected by aging [22]. Collagen is responsible for skin elasticity, elastin contributes to the elasticity of connective tissues, and tyrosine is mainly responsible for skin pigmentation.

Pure surfactants showed good anti-collagenase activity, especially C_12_PAM and LAM. At the tested concentration (35.6 µg/mL), their enzymatic inhibition (Figure 4a), ranged from 50 to 61.1%, similar to that found for the positive control EGCG (54.2%). This result is similar to that found in our previous study, where Gemini-arginine surfactants reached collagenase inhibition between 40 and 50% [5]. Blank zein nanoparticles (BZNps) did not show any activity against this enzyme, and the loaded nanoparticles presented similar values to the surfactants in solution (*p* > 0.05), indicating that they kept their inhibitory effect. This aspect can be important, for instance, during the second phase of wound healing to regulate the deposition of proteins during the fibroblast proliferation phase, promoting its modeling. This is also another important aspect to be explored in alternative wound care applications.

**Figure 9:**
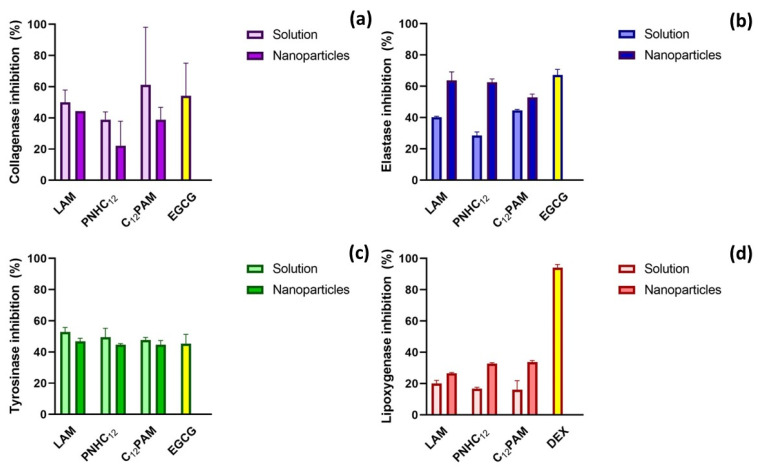
Inhibition of collagenase (**a**), elastase (**b**), tyrosinase (**c**), and lipoxygenase (**d**) by LAM, PNHC_12_, C_12_PAM, PANHC_12_ and their corresponding NPs. EGCG: epigalocatequin-3-galate; DEX: dexamethasone.

The inhibition of elastase was far more evident. For all the surfactants, the nanoencapsulation boosted its inhibitory effect. For instance, the nanoparticles containing PNHC_12_ doubled its inhibitory effect (62.5%) compared to its solution (28.5%) (Figure 9b). C_12_PAM presented the most similar activity between its solution and nanoparticles. The positive control (EGCG) reached 67.3% of elastase inhibition (Figure 9b), comparable to LAM and PNHC_12_ loaded-zein nanoparticles. This result is far different from that found over the Gemini-arginine surfactants, where the elastase inhibition was limited in the best case to 17% [5].

Tyrosinase inhibition was also evident. Both surfactants´ solutions and nanoparticles were found to have a moderate effect over this enzyme, with inhibition ranging between 44.7 and 52.8%, similar to the inhibition obtained with the positive control EGCG (45.4%) (Figure 9c).

Finally, lipoxygenase was the least affected enzyme, with inhibition values ranging from 16.1 to 20.1% for the solutions and 26.5 to 33.8% for the nanoparticles containing the surfactants (Figure 9d). Therefore, despite the limited activity, the nanoparticles improved the anti-inflammatory response of the surfactants upon the inhibition of lipoxygenase.

Hence, anti-enzymatic activities were identified to support the use of surfactants in the treatment of infectious diseases, where tissue disorders are commonly found. While collagenase and elastase inhibition may be helpful in maintaining and stimulating the resistance and elasticity of the skin and also in the repairing of skin, tyrosinase modulation is important to uniform the skin pigmentation, while the inhibition of lipoxygenase may control the inflammatory response. The activities found may be useful in repairing connective tissue or during the skin remodeling stages, for instance, treating hypertrophic and keloid lesions, based on their anti-inflammatory properties.

### 2.6. Molecular Docking Results

**Figure 10:**
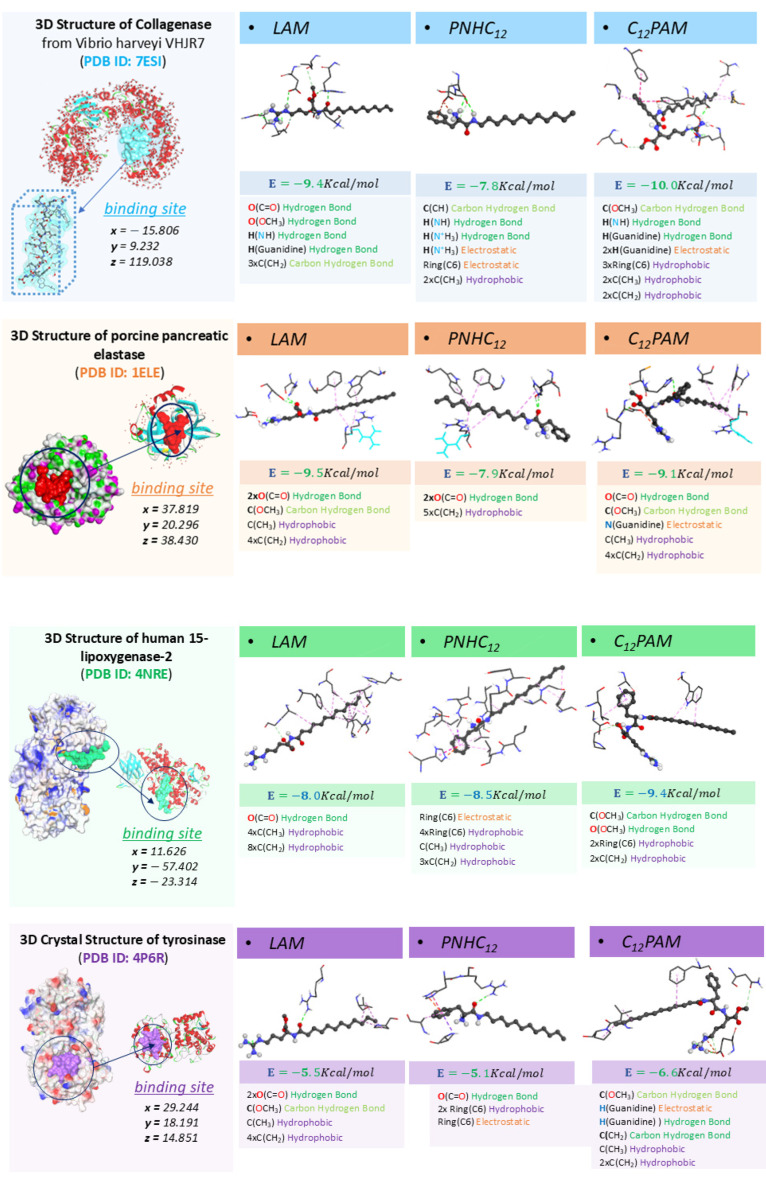
Molecular docking studies showing the various interaction types observed in the contact modes between the surfactant molecules (LAM, PNHC_12_, and C_12_PAM) and the protein pocket of collagenase (PDB ID: 7ESI), elastase (PDB ID: 1ELE), lipoxygenase (PDB ID: 4NRE), and tyrosinase (PDB ID: 4P6R) receptors.

The molecular docking experiments with surfactants (LAM, C_12_PAM, PNHC_12_) for four enzymes, lipoxygenase, collagenase, elastase, and tyrosinase, are really useful for their binding affinities and possible inhibitory mechanisms. It is an insight that allows us to specifically link this theory to experimental data and, thus, to realize the impact of molecular structure, docking scores, and enzyme inhibition relationship (Figure 1 and Figure 10).

For lipoxygenase, it was observed that C_12_PAM contributed with a strong binding strength of −9.4 kcal/mol. The high affinity is due to hydrophobic and hydrogen bonding interactions. PNHC_12_ also exhibited strong inhibitory effects, with a binding energy of −8.5 kcal/mol. The binding energies of LAM were also significant but weaker, −8.0 kcal/mol. The correlation between the experimental findings and the scores yielded from the docking studies indicates that C_12_PAM might serve as a better inhibitor, even though the variances in activity are not much pronounced.

Docking studies for collagenase indicate that C_12_PAM also exhibited the highest binding affinity, with a docking score of −10 kcal/mol followed by LAM with −9.4 kcal/mol. This strong binding is probably attributed to the guanidine groups, which exert strong electrostatic forces. The interaction energy calculated for PNHC_12_ was −7.8 kcal/mol, still enough to suggest standard binding. These observations are reinforced by the experimental results; it was observed that, in solution, C_12_PAM has a slightly stronger inhibition of the enzyme than LAM and PNHC_12_ (61%, 50%, and 44%, respectively). The agreement of the molecular docking and the in vitro results indicates that C_12_PAM and LAM are effective inhibitors of this enzyme in an aqueous solution, while PNHC_12_ is less effective for collagenase.

The binding affinities for elastase indicated by the docking scores showed that LAM has the strongest interaction at −9.5 kcal/mol, followed by C_12_PAM at −9.1 kcal/mol and PNHC12 at −7.9 kcal/mol. These values suggest that these surfactants interact with this enzyme. Experimental results also indicate that LAM is the surfactant with the highest inhibitory activity; however, it was observed that the inhibition percentage of PNHC_12_ was higher than that of C_12_PAM.

According to the tyrosinase docking study, C_12_PAM is the surfactant that exhibited the strongest binding affinity at −6.6 kcal/mol, primarily due to hydrogen bonding and several hydrophobic interactions with the enzyme. LAM showed a lower binding affinity at −5.5 kcal/mol, while PNHC_12_ had the weakest interaction at −5.1 kcal/mol, likely forming weak noncovalent bonds. Despite these differences in binding affinities, the experimental data revealed that all three surfactants exhibited similar inhibitory activity (55%). In general, experimental data generally align with molecular docking results, as seen in the case of lipoxygenase, collagenase, and elastase. This correlation suggests that stronger binding interactions, such as hydrogen bonding and hydrophobic interactions enhance enzyme inhibition. However, somewhat unexpectedly, experimental results did not agree with the docking scores. This discrepancy suggests that inhibition is not solely dependent on binding affinity. Other factors, such as enzyme conformation changes or indirect interactions, may also play a role in the inhibition mechanism, explaining why the experimental results do not directly correlate with docking predictions. Some aspects of enzyme–ligand interactions might not be fully captured in the docking model, accounting for the observed paradox.

The study highlights the importance of molecular structure, specifically the head group type and alkyl chain length, in the enzyme inhibition ability of surfactants. C_12_PAM is the most effective enzyme inhibitor due to its strong electrostatic, hydrogen bonding due to the guanidine group and the two amide bonds and hydrophobic interactions. LAM also shows good potential for enzyme inhibition and performs well in practical applications. In contrast, PNHC_12_, while showing some inhibitory potential, may require structural modifications to improve its effectiveness. Notice that this last compound does not have a guanidine group in their chemical structure.

The obtained results highlight that formulating surfactants into nanoparticles can significantly affect their inhibitory activity against enzymes. For elastase and lipoxygenase, the nanoencapsulation of these surfactants in zein NPs increases their enzymatic activity, while for collagenase the encapsulation reduced their activity, and for tyrosinase both Nps and pure surfactants showed similar anti-enzymatic activity.

In our previous publication, we demonstrated that surfactants like LAM, C_12_PAM, and PNHC_12_ interact with zein to form nanoparticles through various interactions, including hydrogen bonding and hydrophobic interactions [3]. The hydrophobic alkyl chain of PNHC_12_ in nanoparticles is strongly involved with the zein protein, limiting its availability for hydrophobic interactions with the enzyme’s active site. Moreover, some functional groups in the molecular structures of these surfactants, such as the guanidine group in LAM and C_12_PAM and the primary amine in PNHC_12_, may become less available for enzyme interactions as they are involved in stabilizing the nanoparticle structure. Despite its guanidine head group, the amine and the hydrophobic groups remain somewhat available for enzyme interactions; the strong involvement of these are functional groups in stabilizing the nanoparticles, giving rise to a reduction in the activity against some enzymes. These features can explain the cases in which the enzymatic activity of surfactants decreases when encapsulating in NPs.

### 2.7. Cytotoxicity

The surfactants tested were found to be very potent in the biological activities aforementioned. However, the potential applications of these cationic molecules depend on their toxicity against mammalian cells. Recently we have demonstrated that nanoencapsulation was a suitable strategy to reduce the hemolytic activity of these compounds, leaving the antimicrobial activities unaltered [3]. To complete this study, in this work, the cytotoxicity of surfactants in solution and loaded to zein nanoparticles against two representative skin cell lines associated at the epidermis and dermis layers of the skin was determined. In addition, the effect of blank nanoparticles and the vehicle (solution of water/ethanol used to prepare both the pure surfactants and the NPs) was also compared.

**Figure 11:**
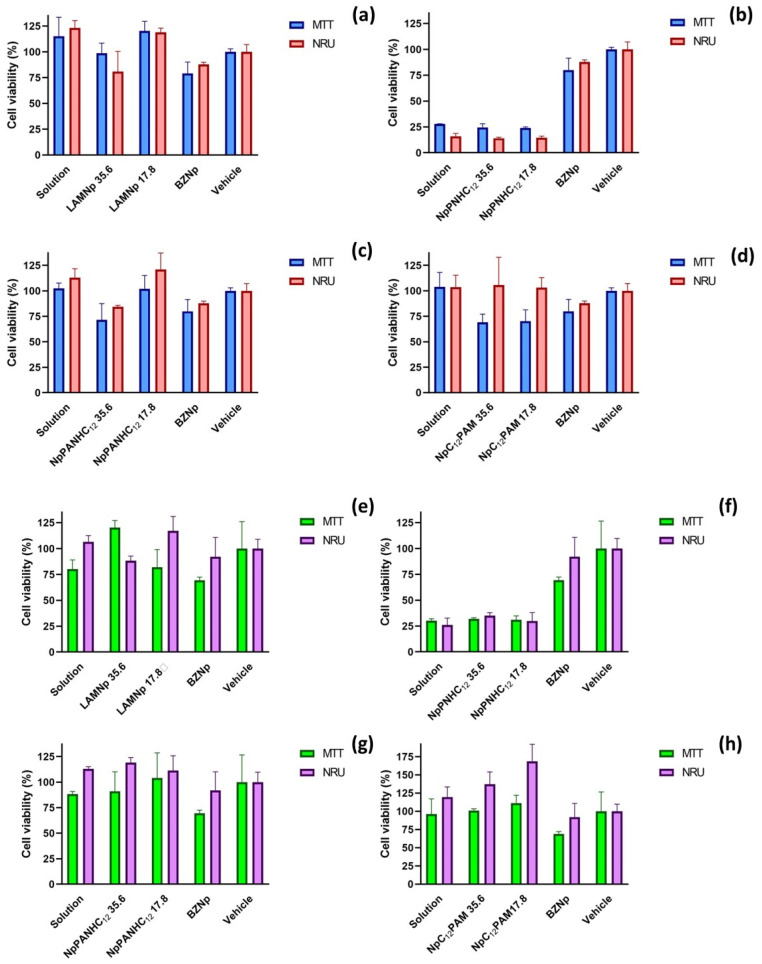
Cellular viability with LAM, PNHC_12_, PANHC_12_, and C_12_PAM in solution (at 35.6 and 17.8 µg/mL) and loaded to zein nanoparticles (at 35.6 and 17.7 µg/mL) over murine Swiss albino fibroblasts (3T3 cell line) (**a**–**d**) and immortal human keratinocytes (HaCaT cell line) (**e**–**h**).

The cytotoxicity was assessed by the colorimetric methods (MTT and NRU assays) over murine fibroblasts (3T3 cell line) and immortal human keratinocytes (HaCaT cell line) (Figure 11). Cells in the absence of any treatment were considered the negative control (100% of viability). The cytotoxic responses were very similar between the used methods, despite the different mechanisms of action, either upon the retention of the dye in the lysosomes for lives cells (NRU method) [23] or the measurement of the reduction of MTT to formazan crystals by metabolically active cells (MTT method) [24]. When fibroblasts were considered (3T3 cell line), cell viabilities higher than 100% were found for all the surfactants in solution, except for the PNHC12 derivative, with cell viabilities of 26% (MTT) and 15% (NRU). A similar trend was found using keratinocytes (HaCaT cell line), although this epithelial cell line seems to be more sensitive to the endpoint method, with MTT values always lower to those obtained with the NRU method (cell viability values around 75 and 100%, respectively). The PNHC12 derivative reported cell viabilities of 25% (MTT) and 20% (NRU) on this cell line.

The surfactants in solution presented acceptable cytotoxic levels, while its nanoencapsulation did not alter this pattern, except for PNHC_12_ in which the cytotoxic effect was found very evident in both solution and nanoencapsulated forms (Figure 11). This aspect might be related to its aggregation behavior, such as that observed in the microbiological results when it lost its activity after nanoencapsulation (Figure 4 and Figure 5). This aspect, together with the high cytotoxicity found, discourages its usage in some pharmaceutical applications. However, the other systems, LAM, PANHC_12_, C_12_PAM and their NPs, exert antimicrobial activity against some of the microorganisms tested at limited and acceptable cytotoxicity levels (Figure 10).

### 2.8. Ecotoxicity Assessment

In order to assess the environmental safety of the investigated cationic surfactants, both in aqueous solution and encapsulated in zein nanoparticles, the standard aquatic toxicity test against *Vibrio fisheri* luminescent bacteria was applied. The comparison between the aquatic toxicity of the surfactant in solution and encapsulated in zein nanoparticles allows one to determine the effect of the incorporation in the NPs on their ecotoxicity. The results of aquatic toxicity expressed as EC_50_ values, i.e., the amount of surfactant necessary to reduce the light emission of bacteria by 50%, are given in Table 2.

The concentration values of these amino acid-based surfactants that reduced bacterial luminescence to 50% in an aqueous solution are very similar between them, ranging from 0.9 to 2.3 mg/L (Table 2). These findings suggest that the common hydrophobic group present in all compounds, i.e., a 12-C aliphatic chain, plays an essential role in aquatic toxicity. In contrast, the nature of the polar group appears to exert a relatively minor influence. The surfactant with an arginine amino acid in the polar head, LAM, is slightly less toxic than the phenylalanine derivative, PNHC_12_, due to the greater hydrophobicity of the amino acid phenylalanine compared to arginine. In the case of surfactants with two amino acids in the polar group, the double-charged surfactant, PANHC_12_, was found to be more toxic than the single-charged surfactant, C_12_PAM. This can be attributed to greater solubility and adsorption capacity within the bacterial membrane of the former. The toxicity values of these surfactants in solution are consistent with previously published values for amphiphilic compounds with the same alkyl chain length as choline-derived amphiphilic ionic liquids [25]. Furthermore, these values align with data previously obtained for LAM. A comparison of the EC_50_ values of surfactants in solution with those of surfactants formulated in zein nanoparticles indicates that the aquatic toxicity of amino acid-based surfactants tends to decrease when incorporated into the nanoparticles. The extent of toxicity reduction is determined by the specific type of surfactant in question. The reduction in toxicity for surfactants comprising a single amino acid in the polar group, namely LAM and PNHC_12_, has been observed to be in the range of 2.5 to 3 times. In the case of the surfactant with two amino acids and two positive charges in the polar head, PANHC_12_, the reduction was relatively minor. This could be attributed to the higher positive charge density of the nanoparticle, as evidenced by the zeta potential data (Table 1), which results in less charge shielding and a higher capacity to exert a toxic effect. However, in the case of the surfactant with two amino acids in the polar group and a single charge, C_12_PAM, the reduction is highly significant, and no toxic effects were observed at the maximum concentration tested. Therefore, it is evident that encapsulation of surfactants in zein nanoparticles results in a notable reduction in their ecotoxicity. Similar results were obtained by Luis et al., as these authors found that when eugenol or/and gallic acid were encapsulated into zein nanoparticles, a significant increase in the LC 50 values against the aquatic biomarker (*Artemia salina*) was observed [26]. These findings indicate that zein nanoparticles can have a protective effect against the ecotoxicity of antimicrobial compounds, such as cationic surfactants.

## 3. Materials and Methods

### 3.1. Materials

Arginine, arginine methyl ester, 3-[4,5-dimethylthiazol-2-yl]-2,5 diphenyl tetrazolium bromide (MTT), neutral red uptake (NRU), menadione, RPMI 1640 and MOPs were purchased from Sigma-Aldrich (St. Louis, MO, USA). Mueller Hinton broth (MHB) and agar (MHA) were purchased from Difco Laboratories (Detroit, MI, USA) and HIMEDIA (Mumbai, India), respectively. Phosphate-Buffer Solution (PBS), sodium hydroxide (NaOH), and HCl were acquired from Merck (Darmstadt, Germany). The cationic surfactants used in this work have been prepared following the procedure described in our previously published paper [3].

### 3.2. Nanoparticles Preparation and Characterization

The nanoparticles were prepared by nanoprecipitation, according to our previous study [3]. Zein (at 0.0178% *w*/*v*) and each surfactant (at 5% *w*/*w*, surfactant/ polymer) were dissolved together in 10 mL of ethanol 70% *v*/*v*, followed by the addition of ultrapure water under constant stirring, obtaining the colloidal dispersion. A formulation containing more concentrated zein and surfactants was also prepared and used in the antibiofilm assays.

### 3.3. Antioxidant Activity

The DPPH scavenging assay was used to access the antioxidant activity. The assay is based on the consumption of the DPPH radical. Nanoparticles containing the surfactants at 35.6 µg/mL were diluted in methanol at different concentrations. Solutions of pure surfactants in methanol at different concentrations were also prepared. Aliquots of 30 µL of each sample solution were added to 270 µL of DPPH methanol solution (40 μg/mL) and kept in the dark for 30 min. The blanks were prepared using 30 µL of each tested solution and 270 µL of pure methanol. The absorbance of DPPH in methanol at the initial concentration of the test and blank samples were measured after 30 min and 24 h in a microplate UV-reader (Biotek) at λ = 517 nm. The antioxidant activity (in %) was calculated according to the following Equation (1):

(1) $$AA\left(\%\right)=\frac{A_{1}-A_{2}}{A_{1}}\times 100$$

where A_1_ is the absorbance of the DPPH solution in methanol and A_2_ is the absorbance of the tested solution after reacting with DPPH.

For the calculation of the IC_50_, a linear fitting model was used, adjusting the linear value (y) to 50.

### 3.4. Biocidal Activity in Suspension

The minimum biocide concentration was accessed for the bacteria (MBC) and yeasts (MFC) in order to check their sensibility to the surfactants in solution and the respective nanoparticles. The American Type Culture Collection (ATCC) *Bacillus subtilis* ATCC 6633, *Staphylococcus aureus* ATCC 29213, *Acinetobacter baumanniii* ATCC 19606, *Pseudomonas aeruginosa* ATCC 27853, *Staphylococcus epidermidis* ATCC 12228, *Escherichia coli* ATCC 25922, *Listeria monocytogenes* ATCC 15313, *Enterococcus faecalis* ATCC 29212, *Candida albicans* ATCC 90028, *Candida jadinni* ATCC 60459, *Candida rugosa* ATCC 10571, *Candida glabrata* ATCC 66032, *Candida parapsilosis* ATCC 22019, *Candida tropicalis* ATCC 7349, *Candida auris* ATCC 21092, and *Candida albicans* ATCC 10231 were the bacteria and yeast used.

First, the minimum inhibitory concentration (MIC) was determined in a 96-well microplate, according to CLSI (2018), varying the concentration of the tested solutions between 35.6 and 2.2 µg/mL. Upon the MIC identification [3], aliquots from that well and two wells immediately above were seeded on MH or SB agar plates and incubated for 24 h. MBC or MFC were defined as the lowest concentration capable of eliminating 99.9% of the microorganisms, identified by the lack of colonies grown on the agar surface.

### 3.5. Antibiofilm Activity

The antibiofilm activity of these formulations were evaluated using two biofilm-producing strains, according to the MTT method [5]: MRSA ATCC 43300 and *Candida albicans* ATCC 90028. Pre-cultures were prepared from a frozen aliquot in MHA (Mullen Hinton agar) at 37 ± 1 °C for 24 h. The inoculum was prepared by suspending 3–4 colonies in lysogeny broth (LB) supplemented with glucose (1%), resulting in a concentration equivalent to 0.5 in the MacFarland scale (~1.5 × 10^8^ CFU/mL). The candida strains were cultured in filter-sterilized RMPI 1640 buffered with 0.165 M MOPS (pH 7.2) at 30 ± 2 °C. This inoculum was adjusted to 1.5 × 10^6^ CFU/mL (equivalent to 0.5 in the McFarland scale).

Biofilms were grown in polystyrene 96-well microtiter plates. For mature biofilm formation, a volume of 200 μL of bacterial or yeast suspension was added to the wells and plates were incubated at 37 °C for 24 h in darkness. Then, non-adherent cells were removed by washing the wells with phosphate-buffered saline (PBS) and 200 μL of the corresponding testing surfactants solution or nanoparticles in serial dilutions were added to each well and incubated again at 37 °C for 24 h. After this period, wells were carefully rinsed with phosphate-buffered saline and the biofilms were stained with MTT/1 mg/mL in PBS) and 1 mM menadione (diluted in acetone) and incubated for 3 h. After this period, the supernatant was removed and the biofilms were dissolved in pure DMSO. The absorbance value was recorded at λ = 570 nm in a Microplate reader (Biotek^®^, Winooski, VT, USA). The biofilm viability was determined according to Equation (2):

(2) $$\%Viability=100-\left(\frac{O.D.SS-O.D.sample}{O.D.SS}\times 100\right)$$

where O.D.SS is the optical density of sterile saline solution (negative control group) after background correction and O.D. sample is the optical density of the testing samples after background correction.

### 3.6. Enzymatic Activities

#### 3.6.1. Collagenase Inhibition Assay

Collagenase inhibition was carried out following the method proposed by Thring et al. [27]. An aliquot of 40 μL of surfactant solution and nanoparticle formulations (35.6 µg/mL) was used in the test. Collagenase from *Clostridium histolyticum* (ChC) was dissolved in tricine buffer (50 mM, pH 8.0) immediately before use at an initial concentration of 0.35 U/mL.

N-[3-(2-furyl) acryloyl]-Leu-Gly-Pro-Ala (FALGPA) was dissolved in the kit buffer (1:1.5) following the supplier’s information. The tested samples were incubated with the enzyme for 15 min, and the substrate was added to obtain a final reaction mixture containing tricine buffer, 0.8 mM FALGPA, 0.1 U of ChC and around 0.712 μg of each cationic amphiphile. Ultrapure water was used as a negative control and an EGCG as a positive control (equivalent to 25 µg prepared in the same vehicle). The absorbance at λ = 345 nm was measured immediately after the substrate addition and continuously during 20 min in kinetic mode using an Elx800 Microplate Reader Biotek^®^ (Winooski, VT, USA). The collagenase inhibitory activity of each sample was determined in triplicate, according to the following Equation (3):

(3) $$Collagenase\;inhibitory\;activity\left(\%\right)=\frac{A_{1}-A_{2}}{A_{1}}\times 100$$

where A_1_ and A_2_ represent the absorbance in the absence and presence of sample, respectively.

#### 3.6.2. Elastase Inhibition Assay

The elastase inhibitory activity was determined following the method described by Thring et al. [27]. Solutions containing the cationic surfactants and those containing the nanoparticles were used (concentration of cationic surfactant was always 35.6 µg/mL). First, a stock solution of porcine pancreatic elastase (3.33 mg/mL) in sterile ultrapure water was prepared. The substrate N-succinyl-N-succinyl- (Ala) 3-p-nitroanilide was dissolved in Tris-HCl buffer solution (0.2 mM and pH 8.0). The samples (40 µL) were then diluted and incubated together with the enzyme for 15 min prior to the substrate addition. The negative control was sterile ultrapure water, while EGCG (250 μM or 0.114 mg/mL) was used as the positive control. The absorbance at λ = 410 nm was measured immediately after the substrate addition and continuously during the next 2 h using an Elx800 Microplate Reader (Biotek^®^, Winooski, VT, USA). The elastase inhibitory activity was evaluated from the release of p-nitroaniline, revealing the substrate proteolysis and subsequent coloring. The anti-elastase activity (in %), determined in triplicate, was calculated according to Equation (4):

(4) $$Elastase\;inhibitory\;activity\left(\%\right)=\frac{A_{1}-A_{2}}{A_{1}}\times 100$$

where A_1_ and A_2_ represent the absorbance in the absence and presence of the testing samples, respectively.

#### 3.6.3. Tyrosinase Inhibition Assay

The tyrosinase inhibitory activity was evaluated by determining the proportion of dopachrome formation, using L-DOPA as the substrate according to the method described by [28] with some adjustments. Briefly, in a 96-well microplate, aqueous tyrosinase solution (40 μL, 200 units/mL), phosphate buffer (pH 6.8, 80 μL), and finally 25 μL of each tested compound were added (surfactants solutions and nanoparticle formulations at 35.6 µg/mL and EGCG at 1000 µg/mL positive control), mixed, and pre-incubated for 10 min at 37 °C. Then, 40 μL of L-DOPA (15 mM) was added, and the mixture was incubated again for 30 min at 37 °C. The quantity of dopachrome was measured at λ = 492 nm ( Kasuaki^®^ DR-200BS-NM-BI microplate reader, Tokyo, Japan). The percentage of tyrosinase inhibition was calculated using the next Equation (5):

(5) $$Tyrosinase\;inhibitory\;activity\left(\%\right)=\frac{A_{c}-A_{s}}{A_{c}}\times 100$$

where A_c_ is the absorbance of the control and A_s_ is the absorbance of the testing sample.

#### 3.6.4. Lipoxygenase Inhibition Assay

The lipoxygenase (LOX) inhibitory activity was performed according to the method used by Ksiksi et al. [29] with some modifications. Briefly, a mixture of sodium borate buffer (1 mL, 0.1 M, pH 8.8), lipoxygenase soybean (10 µL, at 8000 U/mL), and the test substances (surfactants solutions and nanoparticles at 35.6 µg/mL) (10 µL) was incubated in a 1 mL quartz cuvette at room temperature for 15 min. The reaction was initiated by adding a linoleic acid substrate (10 µL, 51 mM). The absorbance of the resulting mixture was measured at λ = 234 nm (Perkinelmer® Lambda 35, Waltham, MA, USA) 3 times with a 1 min interval. Dexamethasone (DEX) at 200 µg/mL was used as positive control. The inhibition of LOX was assessed using the following Equation (6):

(6) $$Lipoxygenase\;inhibitory\;activity\left(\%\right)=\frac{A_{1}-A_{2}}{A_{1}}\times 100$$

where A_1_ and A_2_ represent the absorbance in the absence and presence of the test sample, respectively.

### 3.7. Molecular Docking 

To elucidate the binding modes of the evaluated surfactants within the target enzymes, molecular docking simulations were performed using AutoDock Vina [30]. The study used the crystal structure of collagenase from *Vibrio harveyi* (PDB ID: 7ESI) [31], the three-dimensional structure of porcine pancreatic elastase (PDB ID: 1ELE) [32], the crystal structure of lipoxygenase (PDB ID: 4NRE) [33], and the three-dimensional structure of tyrosinase (PDB ID: 4P6R) [34]. To correct for ionization and tautomeric states of amino acid residues, polar hydrogen atoms were added to the protein structures [35]. The binding sites on collagenase, elastase, and hyaluronidase were defined using the AutoGrid step, referencing the original ligands. A grid box with dimensions (x, y, z) = (16, 38, 16) was selected to encompass the binding sites of interest. Default parameters were utilized in AutoDock Tools 1.5.4 [36].

The surfactant ligands were drawn using ChemDraw 20.1.1, and to identify the most stable conformations, the ligand geometries were optimized using the Molecular Mechanics Force Field (MMFF94). Subsequently, the ligand and target protein files were converted to the PDBQT format to ensure compatibility with AutoDock Vina 1.2.0 for docking purposes. The resulting protein-ligand complexes were analyzed for interactions using Discovery Studio Client software (version 22.1), allowing for a comprehensive understanding of the binding interactions between the surfactants and the enzymes.

### 3.8. Cytotoxicity Assays

Two representative skin cell lines, immortal human keratinocytes (HaCaT cell line) and murine Swiss albino fibroblasts (3T3 cell line), were obtained from Celltec, University of Barcelona. The cells were grown in high glucose DMEM (4.5 g/L glucose) supplemented with 10% (*v*/*v*) FBS, 2 mM L-glutamine, and antibiotics (100 U/mL of penicillin and 100 μg/mL of streptomycin) at 37 °C under 5% CO_2_. Cells were routinely cultured in 75 cm^2^ culture flasks and split using trypsin-EDTA when approximately 80% of confluence was reached.HaCaT cells (1 × 10^5^ cells/mL) and 3T3 cells (5 × 10^4^ cells/mL) were grown at the defined densities into the central 60 wells of a 96-well microplate. Cells were incubated under 5% CO_2_ at 37 °C for 24 h. The medium was then removed, and the cells were incubated with the surfactants and nanoparticles (100 μL), previously diluted 1:1 (*v*/*v*) in DMEM supplemented with 5% FBS, reaching a concentration of 35.6 and 17.8 µg/mL for the nanoparticles, while the surfactants solutions were assayed at the higher concentration. Two different colorimetric methods for quantification were used, as follows.

#### 3.8.1. The Neutral Red Uptake (NRU) Assay

The neutral red uptake (NRU) assay is based on the accumulation of the dye in the lysosomes of viable cells [23]. After 24 h of incubation with the corresponding formulations, the medium was removed and the cells were incubated for 3 h with the NRU dye solution (50 μg/mL) dissolved in medium (without phenol red and FBS). Cells were washed once with sterile PBS and treated with 100 μL of a solution containing 50% ethanol absolute and 1% acetic acid in distilled water to extract the non-adhered dye. Plates were then placed in a microtiter-plate shaker for 5 min at room temperature to help the total dissolution. Then, the absorbance of the resulting solutions was measured at 550 nm using a Bio-Rad 550 microplate reader (Bio-Rad, Hercules, CA, USA).

#### 3.8.2. MTT Assay

2,5 Diphenyl-3, -(4,5-dimethyl-2-thiazolyl) tetrazolium bromide (MTT) can be reduced on living cells from yellow tetrazolium salt to insoluble purple formazan crystals [24]. Once the cells were incubated for 24 h with the corresponding formulations, the medium was removed, and cells were incubated for 3 h with 100 μL of MTT in PBS (5 mg/mL) diluted 1:10 in culture medium (without phenol red and FBS). After medium removal, 100 μL of DMSO was added to dissolve the purple formazan crystals. Following agitation, the absorbance of the extracted solution was measured at the same conditions as in Section 3.8.2.

### 3.9. Ecotoxicological Assessment: Vibrio fisheri Luminescence Reduction Test

The *V. fisheri* bioluminescence assay [37] was employed to evaluate the ecotoxicity of amino acid-based surfactants, both in solution and when entrapped in zein nanoparticles. Following exposure of the luminescent bacteria to the toxic compound, a decrease in light emission was observed. Regression analysis was used to calculate the EC50 values (concentration of surfactant that caused a 50% reduction in the amount of light emitted by the bacteria). The stated toxicity statistics are based on the bacteria being exposed to the surfactant and NP solutions for 15 min at 15 °C.

### 3.10. Statistical Analysis

The results were expressed as mean ± SD. The data were analyzed using ANOVA followed by Tukey’s post-hoc test. A value of *p* < 0.05 was considered statistically significant. Statistical analyses were performed using the software GraphPad Prism version 9.0.

## 4. Conclusions

Biodegradable arginine-phenylaniline surfactants showed potent antimicrobial activity against Gram-positive, Gram-negative bacteria and yeasts, including the worrisome *Candida auris*. Most surfactants maintained their activity after nanoencapsulation in zein nanoparticles. The nanoformulations were found to be very active against the MRSA and *Candida albicans* biofilms, increasing the effect of the surfactants.

The nanoencapsulation of these surfactants enhanced the antioxidant activity. Pure solutions of these surfactants and their NPs exhibit good inhibitory activities over collagenase, elastase, and tyrosinase, key enzymes involved in the tissue repair processes. While collagenase and tyrosinase activities were maintained after nanoencapsulation, elastase and lipoxygenase inhibition were increased. The binding affinities obtained by molecular docking experiments with these surfactants and the four enzymes (lipoxygenase, collagenase, elastase, and tyrosinase) agree with the inhibitory activity experimentally obtained.

The cytotoxicity of surfactants’ solutions and nanoparticles under the concentrations and conditions used was limited and acceptable, with the exception to the formulations containing PNHC_12_. Finally, it was observed that the nanoencapsulation of these compounds reduced their aquatic toxicity.

Therefore, this approach was demonstrated to be a feasible strategy to improve key pharmacological targets found during the skin repairing mechanisms. The obtained results make these surfactants and their NPs promising antimicrobial systems to be used in wound care applications, where a robust antimicrobial activity is required and additional pharmacological activities related to the skin repairing mechanisms are desirable. The systems obtained combined the two aspects and also presented limited cytotoxicity over cell lines representative of skin and connective tissues. Accordingly, they represent promising candidates for wound prevention and treatment.

## Figures and Tables

**Table 1:** Physicochemical characteristics of blank and arginine-phenylalanine-based surfactants loaded to zein nanoparticles.

Formulation	Size (nm) ± SD	pdI	Zeta Potential (mV)
BZNp	194.3 ± 4.8	0.101	+16.5
NpLAM	287.0 ± 4.2	0.048	+29.7
NpPNHC_12_	272.8 ± 13.1	0.052	+24.9
NpPANHC_12_	286.3 ± 5.9	0.046	+44.3
NpC_12_PAM	312.5 ±6.3	0.106	+34.7

**Table 2:** Aquatic toxicity of amino acid-based surfactants both in solution and formulated in zein nanoparticles using the *Vibrio fisheri* luminescence inhibition bioassay. Expressed as EC_50_ concentration, with a 95% confidence interval.

Surfactant	EC_50_ (95% CI) (mg/L)	
	Solution	Nanoparticles
LAM	1.7 (1.3–2.2)	5.4 (2.4–12)
PNHC_12_	0.9 (0.7–1.1)	2.3 (0.3–16)
PANHC_12_	1.7 (1.0–2.9)	2.1 (0.5–8.0)
C_12_PAM	2.3 (0.4–14)	>9

Blank zein NPs did not produce any reduction in luminescence at the maximum concentration tested (50 µg/mL).

## Data Availability

Data are contained within the article.
